# Degron tagging of BleoR and other antibiotic-resistance genes selects for higher expression of linked transgenes and improved exosome engineering

**DOI:** 10.1016/j.jbc.2022.101846

**Published:** 2022-03-18

**Authors:** Shang Jui Tsai, Yiwei Ai, Chenxu Guo, Stephen J. Gould

**Affiliations:** Department of Biological Chemistry, Johns Hopkins University, Baltimore, Maryland, USA

**Keywords:** selectable marker, transgenic, blasticidin, G418, hygromycin, puromycin, zeocin, CD63, extracellular vesicle, antibiotic, AR, antibiotic resistance, CM, chemically defined media, CMV, cytomegalovirus, ecDHFR, *Escherichia coli* dihydrofolate reductase, ER50, estrogen receptor 50, FACS, fluorescence-activated cell sorting, gRNA, guide RNA, HBSS, Hank’s buffered saline solution, HRP, horseradish peroxidase, REE, recombinantly engineered exosome, SCC, single-cell clone, TBST, 50 mM Tris–HCl, pH 8.0, 138 mM NaCl, 2.7 mM KCl, 0.05% Tween-20, UME, unmodified exosome

## Abstract

Five antibiotic resistance (AR) genes have been used to select for transgenic eukaryotic cell lines, with the BleoR, PuroR, HygR, NeoR, and BsdR cassettes conferring resistance to zeocin, puromycin, hygromycin, geneticin/G418, and blasticidin, respectively. We recently demonstrated that each AR gene establishes a distinct threshold of transgene expression below which no cell can survive, with BleoR selecting for the highest level of transgene expression, nearly ∼10-fold higher than in cells selected using the NeoR or BsdR markers. Here, we tested the hypothesis that there may be an inverse proportionality between AR protein function and the expression of linked, transgene-encoded, recombinant proteins. Specifically, we fused each AR protein to proteasome-targeting degron tags, used these to select for antibiotic-resistant cell lines, and then measured the expression of the linked, recombinant protein, mCherry, as a proxy marker of transgene expression. In each case, degron-tagged AR proteins selected for higher mCherry expression than their cognate WT AR proteins. ER50BleoR selected for the highest level of mCherry expression, greater than twofold higher than BleoR or any other AR gene. Interestingly, use of ER50BleoR as the selectable marker translated to an even higher, 3.5-fold increase in the exosomal loading of the exosomal cargo protein, CD63/Y235A. Although a putative CD63-binding peptide, CP05, has been used to decorate exosome membranes in a technology known as “exosome painting,” we show here that CP05 binds equally well to CD63^−/−^ cells, WT 293F cells, and CD63-overexpressing cells, indicating that CP05 may bind membranes nonspecifically. These results are of high significance for cell engineering and especially for exosome engineering.

In the 4 decades since the invention of mammalian cell transgenesis (1), biomedical research has become highly dependent on the creation, analysis, and use of transgenic eukaryotic, and especially mammalian, cell lines (2, 3). During this time, numerous improvements have been made to nearly every aspect of vector design, including choice of transcriptional control regions, introns, polyadenylation sites, mRNA export sequences, translation initiation–promoting sequences, codon utilization, and mode of transgene delivery (4, 5, 6, 7, 8, 9, 10, 11, 12, 13, 14, 15, 16, 17, 18). As a result, there is now a wealth of information on how to create transgenic mammalian cells and especially for the production of recombinant proteins of interest.

We recently reported that the choice of antibiotic resistance (AR) gene can also have a major impact on transgene expression (19). More specifically, we observed that each combination of antibiotic and AR gene/protein establishes a unique threshold of transgene expression below which no cell can survive, suggesting an inverse relationship between (i) the activity and/or stability of each dominant selectable marker protein and (ii) the average level of transgene expression across a population of antibiotic-resistant cell clones. This hypothesis is relevant to mammalian cell transgenesis in general but may be particularly relevant to the production of transgenic mammalian cell lines used for making recombinantly engineered exosomes (REEs).

Exosomes are small secreted organelles (diameter of ∼30–200 nm) that have the same topology as the cell, are highly enriched in a subset of proteins, lipids, and RNAs, and can transmit signals and molecules through an intercellular vesicle trafficking pathway (20). Furthermore, exosomes appear to be generated by a stochastic process that operates across a spectrum of plasma and endosome membranes, leading to pronounced compositional heterogeneity of individual exosomes (20, 21). As a result, failure to maintain high transgene expression in nearly 100% of the cells during the production of exosomes will result in the release of large quantities of unmodified exosomes (UMEs). UMEs are a major problem in exosome engineering, in part because they represent lost yield, but even more because they are difficult if not impossible to separate from REEs, complicating the production and analysis of REEs. Fortunately, the stochastic nature of exosome biogenesis (21) means that the ratio of REEs to UMEs may be increased by high-level expression of the recombinant exosome cargo protein. Under this model, the invention of new AR genes that allow for the rapid and easy selection of high-expressing cell lines has even greater relevance to the specific field of exosome engineering.

Our operating hypothesis predicts that one way to create more restrictive AR genes is to tag them with destabilization domains, or degrons, which are known to target proteins to the proteasome and thereby reduce their steady-state abundance and net activity in the cell (22). Of the previously characterized degrons, those from the estrogen receptor 50 (ER50) and *Escherichia coli* dihydrofolate reductase (ecDHFR) are particularly intriguing, as they can be stabilized in a dose-dependent manner by small molecules (4-hydroxytamoxifen in the case of ER50 and trimethoprim in the case of ecDHFR (23, 24, 25)). Here, we describe the results of degron tagging the five commonly used AR proteins. In the process, we created improved versions of all five AR genes/proteins, culminating in the creation of ER50BleoR. This new marker gene selects for the highest transgene expression of any selectable marker and also drives superior loading of the exosomal cargo protein CD63/Y235A into secreted exosomes. This latter finding raised the possibility that these exosomes would be ideal substrates for surface decoration by CP05-based ligands, which are thought to bind specifically to the extracellular domain of CD63. However, our data indicate that CP05 peptides bind biological membranes independently of CD63.

## Results

### Degron tagging of AR proteins

**Figure 1:**
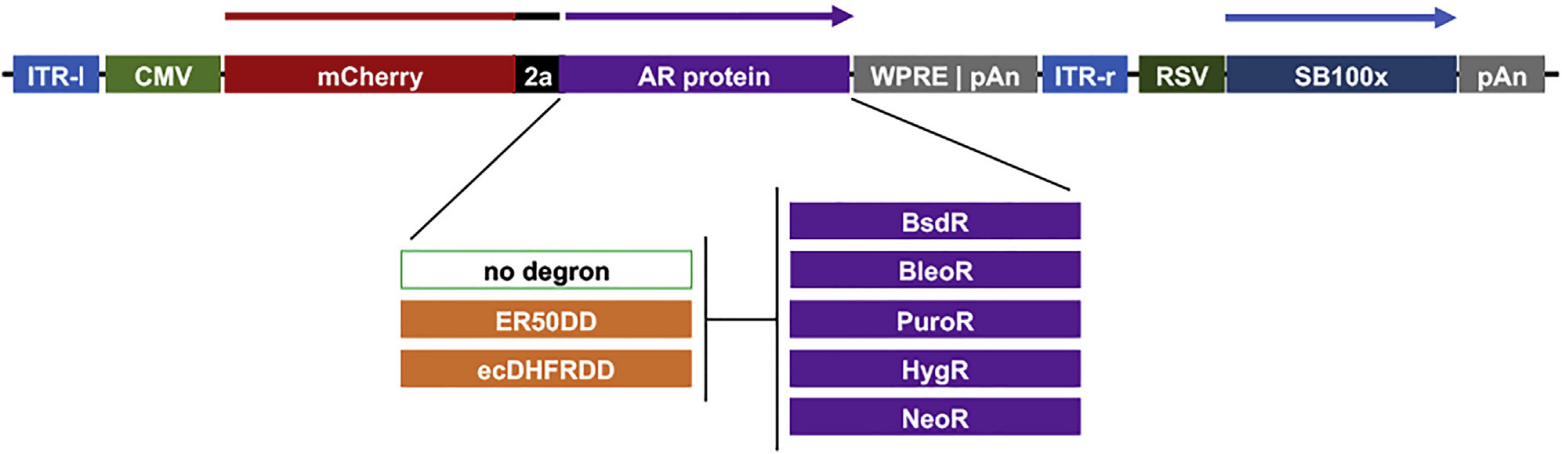
**Line diagrams of AR gene test vectors.** Fifteen distinct Sleeping Beauty transposon–containing vectors were created, each carrying a single transposon–carried gene in which the CMV enhancer/promoter was positioned to drive the expression of a bicistronic ORF encoding (i) mCherry, (ii) a viral 2a peptide, and (iii) an AR protein. These AR proteins included untagged ER50 degron-tagged or ecDHFR degron-tagged forms of the five AR proteins BsdR, BleoR, PuroR, HygR, and NeoR (19). Following transfection of these vectors into 293F cells, expression of the Sleeping Beauty transposase SB100X is driven from the Rous sarcoma virus long-terminal repeat, leading to mobilization of all sequences including and between the inverted tandem repeats, from the vector and into one or more sites in the host cell genome, leading subsequently to expression of mCherry in a manner dependent upon integration site and integrant copy number, both of which can vary dramatically within the population of transgenic cells. AR, antibiotic resistance; CMV, cytomegalovirus; ecDHFR, *Escherichia coli* dihydrofolate reductase; ER50, estrogen receptor 50.

To determine whether destabilization domains could impact the levels of transgene expression, we generated pITRSB-based Sleeping Beauty transposons (12, 19) that use cytomegalovirus (CMV) enhancer/promoter sequences to drive the expression of bicistronic ORFs (Fig. 1). These ORFs encode (i) the fluorescent protein mCherry (26), (ii) an 18 amino acid–long viral 2a peptide (27, 28), and (iii) one of 15 different AR proteins, which correspond to untagged, ER50-tagged, or ecDHFR-tagged forms of the BsdR, NeoR, HygR, PuroR, and BleoR proteins (29, 30, 31, 32, 33). These 15 transposon-carrying vectors were transfected into 293F cells, followed 2 days later by transfer of the transfected cell populations into selective media. Selective media were changed every 3 to 5 days for 2 weeks, resulting in the death of all untransfected cells, as well as all transgenic cell lines that failed to express enough of the AR protein to confer survival and growth in the antibiotic-containing media. At the end of this period, thousands of single-cell clones (SCCs) were apparent, which together represent the range of transgene expression levels generated by each vector. To capture this range, all SCCs from each transfection were pooled to create a single polyclonal cell line. SCCs were generated from all transfections except for one (cells transfected with the ecDHFRBleoR-based vector yielded only a very small number of poorly adherent and poorly growing cells, and the resulting line was unsuitable for further analysis).

The 14 polyclonal cell lines that emerged from this process were expanded for 2 additional weeks in selective media and then interrogated by flow cytometry to measure the mean, median, and individual mCherry expression levels across ∼20,000 cells from each cell line. Consistent with our operating hypothesis, degron-tagged forms of the BsdR, BleoR, PuroR, HygR, and NeoR proteins were selected for higher levels of expression of the linked recombinant protein mCherry (Table 1). However, the results varied significantly between these different AR genes, warranting a more in-depth discussion of each group of antibiotic-resistant cell lines.

**Table 1:** Flow cytometry measurement of mCherry fluorescence brightness (a.u.) of transgenic 293F-derived cell lines

AR protein	Mean	Increase in mean	Median	Increase in median	Mean/median	CV
BsdR	1308	NA	28	NA	47	370
ER50BsdR	6646	5.1×	3424	122×	1.9	140
ecDHFRBsdR	7978	6.1×	4696	168×	1.7	136
BleoR	16,025	NA	13,093	NA	1.2	84
ER50BleoR	37,141	2.3×	33,678	2.6×	1.1	48
ecDHFRBleoR	ND	ND	ND	ND	ND	ND
PuroR	6539	NA	4595	NA	1.4	107
ER50PuroR	10,808	1.7×	8592	1.9×	1.3	77
ecDHFRPuroR	10,898	1.7×	8780	1.9×	1.2	81
HygR	6807	NA	3912	NA	1.7	125
ER50HygR	6532	0.9×	4567	1.2×	1.4	97
ecDHFRHygR	8455	1.2×	5961	1.5×	1.4	99
NeoR	4498	NA	2543	NA	1.8	126
ER50NeoR	5748	1.3×	3828	1.5×	1.5	110
ecDHFRNeoR	5790	1.3×	3852	1.5×	1.5	107

Abbreviations: NA, not applicable; ND, not determined.

### Degron tagging improved blasticidin/BsdR-selected transgene expression by sixfold

Of the five antibiotics commonly used in mammalian cell transgenesis experiments (blasticidin, geneticin, hygromycin B, puromycin, and zeocin), blasticidin kills fastest of all, as addition of blasticidin killed 100% of 293F cells within 48 h. Curiously, when 293F cells were transfected with the transposon carrying the CMV-mCherry-2a-BsdR transgene, we observed relatively little cell death and robust growth of nearly all cells in the population. These results indicate that most of the cells (i) had been transfected, (ii) continued to express the BsdR protein, and (iii) expressed enough blasticidin deaminase enzyme activity to confer blasticidin resistance on the cells, regardless of the level of transgene expression.

**Figure 2:**
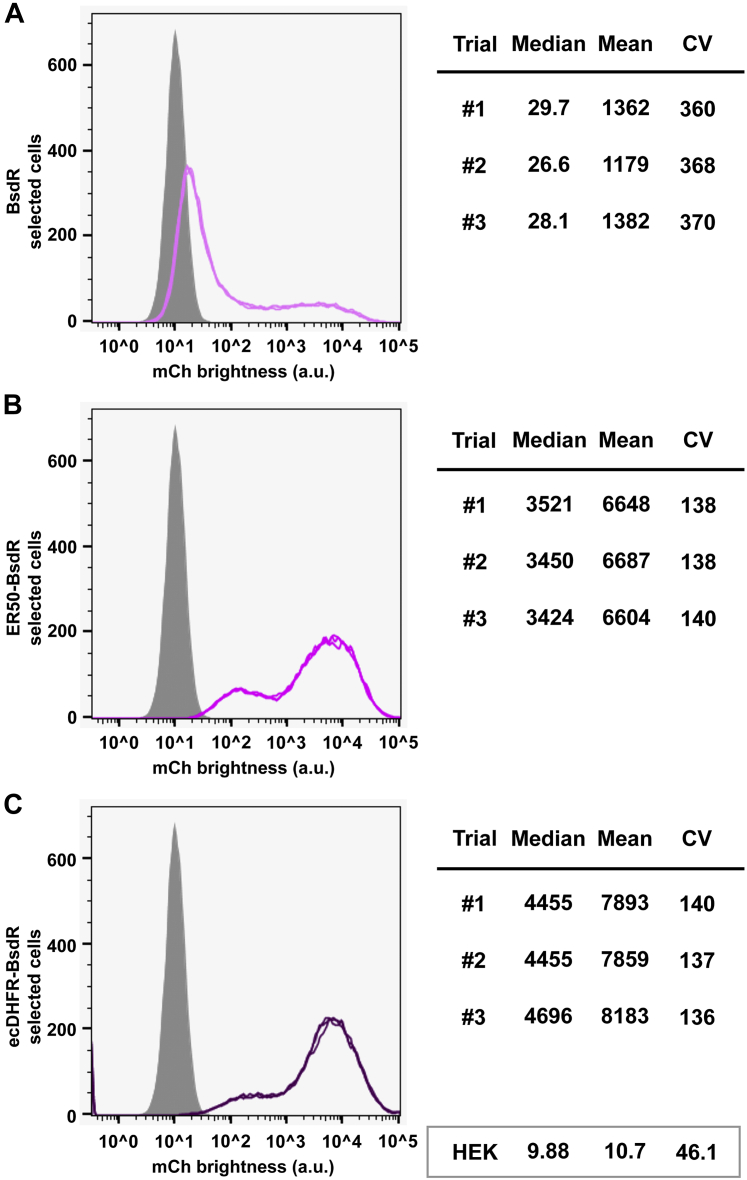
**Degron-tagging BsdR genes results in approximately fivefold higher expression of the linked recombinant protein mCherry.** Flow cytometry measurements of mCherry expression levels (fluorescence brightness, arbitrary units) in polyclonal cell lines that were generated following transfection with transposons carrying (*A*) the untagged BsdR ORF, (*B*) the ER50BsdR ORF, or (*C*) the ecDHFRBsdR ORF. Data are from three technical replicates of each cell line. Approximately 20,000 cells were assayed in each replicate, *gray* shows the background fluorescence of 293F cells, and the median, mean, and CV from each replicate are shown to the *right*. *Light purple* shows mCherry expression in the BsdR cell line data, *medium purple* shows the ER50BsdR cell line data, and *dark purple* shows the ecDHFRBsdR cell line data. Data from 293F cells are shown in the *box* at the *bottom right* of the figure. ecDHFR, *Escherichia coli* dihydrofolate reductase; ER50, estrogen receptor 50.

Consistent with this notion, we found that the blasticidin-resistant cell line resulting from transfection with the BsdR-based vector displayed the lowest level of mCherry expression and the greatest cell-to-cell heterogeneity in mCherry of any cell line tested (Table 1 and Fig. 2*A*). In fact, many of the blasticidin-resistant cells in this polyclonal cell line displayed levels of mCherry fluorescence that were no higher than the background fluorescence of 293F control cells. In contrast to the BsdR-selected cell line, the ER50BsdR-selected cell line displayed mean mCherry expression levels that were, on average, approximately fivefold higher than the BsdR-selected cell line, whereas the ecDHFRBsdR-selected cell line displayed average mCherry expression approximately sixfold higher than the BsdR-selected cell line (Table 1 and Fig. 2, *B* and *C*). The pronounced effect of BsdR degron tagging on transgene expression is evident from a number of perspectives, which include (i) 122-fold higher and 168-fold higher median fluorescence levels, (ii) the threefold lower CV values (∼365), and (iii) the drop in mean/median ratio from ∼45 to ∼2.

### ER50BleoR, the most-restrictive dominant selectable marker

**Figure 3:**
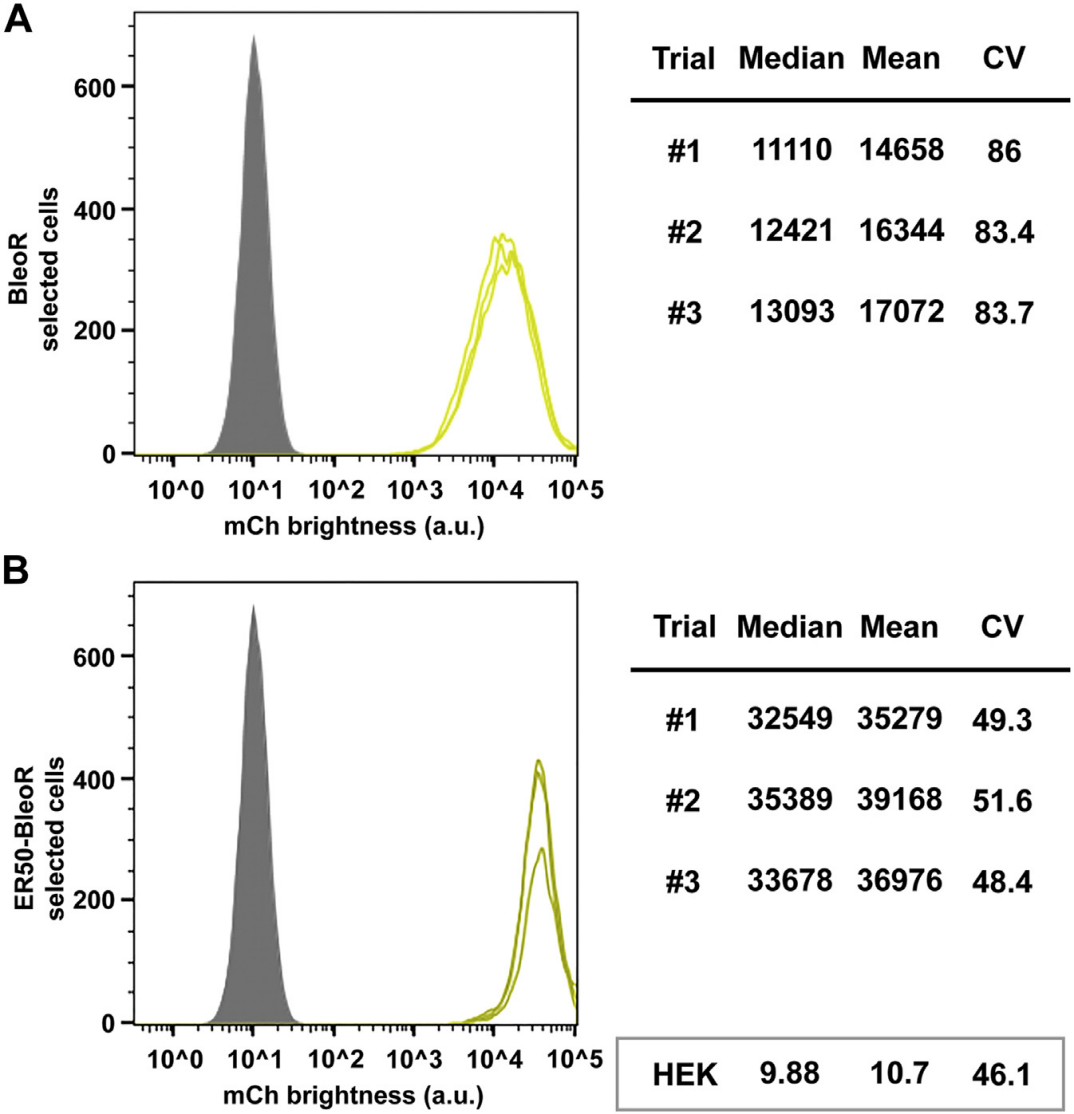
**ER50BleoR selects for twofold higher levels of linked mCherry expression.** Flow cytometry histograms of mCherry expression levels (fluorescence brightness, arbitrary units) in the polyclonal cell lines selected using transposons carrying (*A*) the untagged BleoR ORF and (*B*) the ER50BleoR ORF. Data are shown for three technical replicates of each cell line, involving ∼20,000 independent cell fluorescence measurements for each replicate, with *gray* showing the background fluorescence of 293F cells, and the median, mean, and CV from each replicate are shown to the *right*. *Light chartreuse* shows the BleoR cell line data, whereas *dark chartreuse* shows the ER50BleoR cell line data. Data from 293F cells are shown in the *box* at the *bottom right* of the figure. ER50, estrogen receptor 50.

We previously established that the BleoR gene selects for higher and more homogeneous transgene expression than the BsdR, PuroR, HygR, or NeoR selectable marker genes (19). We observed the same in the present study, as the mean mCherry fluorescence of the BleoR-selected cell line was >10-fold higher than that of the BsdR-selected cell line (Table 1). In perhaps the most important invention of this study, we found that a degron-tagged form of BleoR, ER50BleoR, selected for a further 2.3-fold increase in average mCherry expression relative to the BleoR-selected cell line, making it the most restrictive selectable marker of all (Table 1; Fig. 3, *A* and *B*).

As predicted from our operating hypothesis, this 2.3-fold increase in mean transgene expression led to a further reduction in the cell-to-cell heterogeneity of mCherry expression, shown here by the drop in the CV from 84 to 48. It should, however, be noted that >99% of the cells in both the BleoR and ER50BleoR-selected cell lines expressed mCherry fluorescence that was >1000-fold above background, evidence that both forms of the BleoR gene are quite good at generating cell lines in which nearly every surviving cell expresses a high level of transgene expression. That being said, the superior performance characteristics of the ER50BleoR marker can be seen throughout our data, even in the mean/median ratio that fell to 1.1, just shy of the theoretical optimum of 1. Taken together, these results demonstrate that the ER50BleoR AR gene can be used to rapidly select for polyclonal cell lines that express the very highest levels of linked transgene expression, without the need for isolating and screening large numbers of SCCs. However, if SCCs are needed, selection with the ER50BleoR marker is likely to eliminate all but the highest-expressing clones, reducing the number of clones that need to be screened to identify the highest-expressing cell lines.

### Degron tagging increased PuroR-selected transgene expression by 70%

In our original report on how AR gene choice affected transgene expression, we found that the PuroR and HygR genes yielded the second-highest levels of linked recombinant protein expression, ∼50% that of zeocin-resistant BleoR-derived cell lines, yet significantly higher than cell lines created using the BsdR or NeoR selectable marker genes (19). We observed similar results in the present study, as PuroR-based and HygR-based cell lines expressed nearly identical levels of mCherry fluorescence (6539 *versus* 6807 arbitrary units [a.u.], respectively), a level that was ∼40% the level selected by the BleoR marker (16,025 a.u.), and higher than the levels of transgene expression observed in cell lines selected *via* the NeoR (4498 a.u.) or BsdR (1308 a.u.) markers (Table 1).

**Figure 4:**
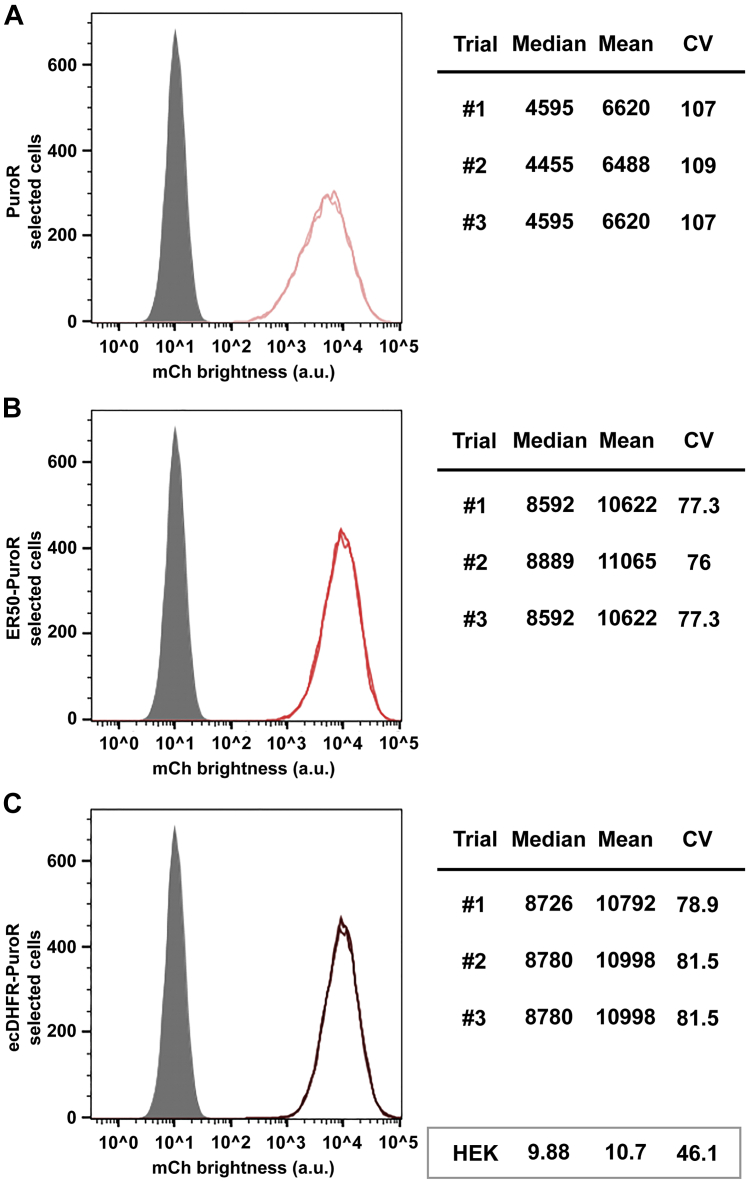
**Degron-tagging PuroR increases linked mCherry expression by 70%.** Flow cytometry measurements of mCherry expression levels (fluorescence brightness, arbitrary units) in polyclonal cell lines selected *via* the (*A*) untagged PuroR ORF, (*B*) ER50BleoR ORF, and (*C*) ecDHFRPuroR ORF. Data are shown for three technical replicates of each cell line, involving ∼20,000 independent cell fluorescence measurements for each replicate, with *gray* showing the background fluorescence of 293F cells, and the median, mean, and CV from each replicate are shown to the *right*. *Light coral* shows the PuroR-selected cell line data, *medium coral* shows the ER50PuroR-selected cell line data, and *dark coral* shows the ecDHFRPuroR-selected cell line data. Data from 293F cells are shown in the *box* at the *bottom right* of the figure. ecDHFR, *Escherichia coli* dihydrofolate reductase; ER50, estrogen receptor 50.

As for the degron-tagged forms of PuroR, they selected for ∼70% higher mean mCherry expression (Fig. 4, *A*–*C*). While the amplitude of this effect was less than we observed for the BsdR and BleoR AR genes, it did lead to reduced heterogeneity of expression, shown here by drop on CV from 107 to ∼80 and a fall in mean/median ratio, from 1.4 to 1.2.

### Degron tagging had only minimal effects on HygR-selected and NeoR-selected transgene expression

**Figure 5:**
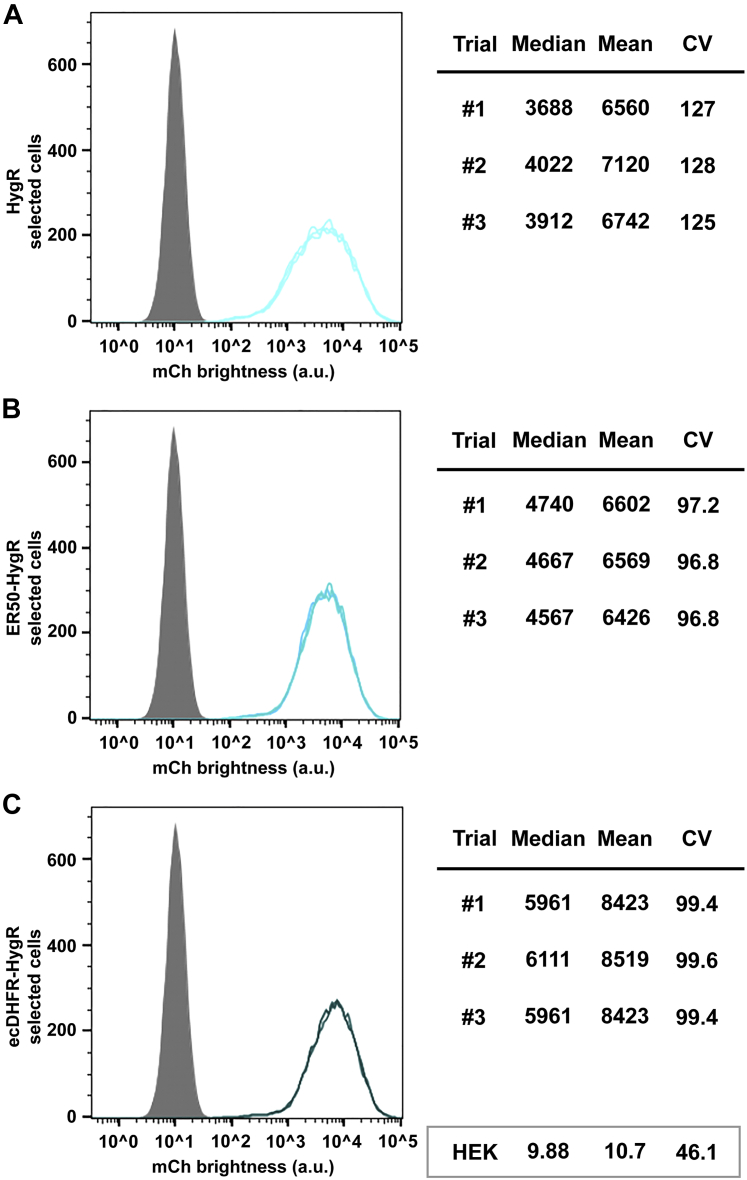
**Degron tagging has only minimal effects on HygR-selected transgene expression.** Flow cytometry measurements of mCherry expression levels (fluorescence brightness, arbitrary units) in the polyclonal cell lines generated following transfection with transposons carrying transgenes expressing the (*A*) untagged HygR ORF, (*B*) ER50HygR ORF, and (*C*) ecDHFRHygR ORF. Data are shown for three technical replicates of each cell line, involving ∼20,000 independent cell fluorescence measurements for each replicate, with *gray* showing the background fluorescence of 293F cells, and the median, mean, and CV from each replicate are shown to the *right*. *Light cyan* shows the HygR-selected cell line data, *medium cyan* shows the ER50HygR-selected cell line data, *dark cyan* shows the ecDHFRHygR-selected cell line data. Data from 293F cells are shown in the *box* at the *bottom right* of the figure. ecDHFR, *Escherichia coli* dihydrofolate reductase; ER50, estrogen receptor 50.

In contrast to BsdR, BleoR, and PuroR, HygR was at most slightly sensitive to degron tagging, if at all. While both degron-tagged forms of the HygR marker resulted in slight increases in the homogeneity of expression, the increase in mean mCherry expression was either undetectable (in the case of ER50HygR-selected cells) or only 24% (in the case of ecDHFRHygR-selected cells) (Fig. 5, *A*–*C*). This was, of course, unexpected, and raised questions about why degron tagging could have a strong effect on some AR proteins but little if any effect on others.

**Figure 6:**
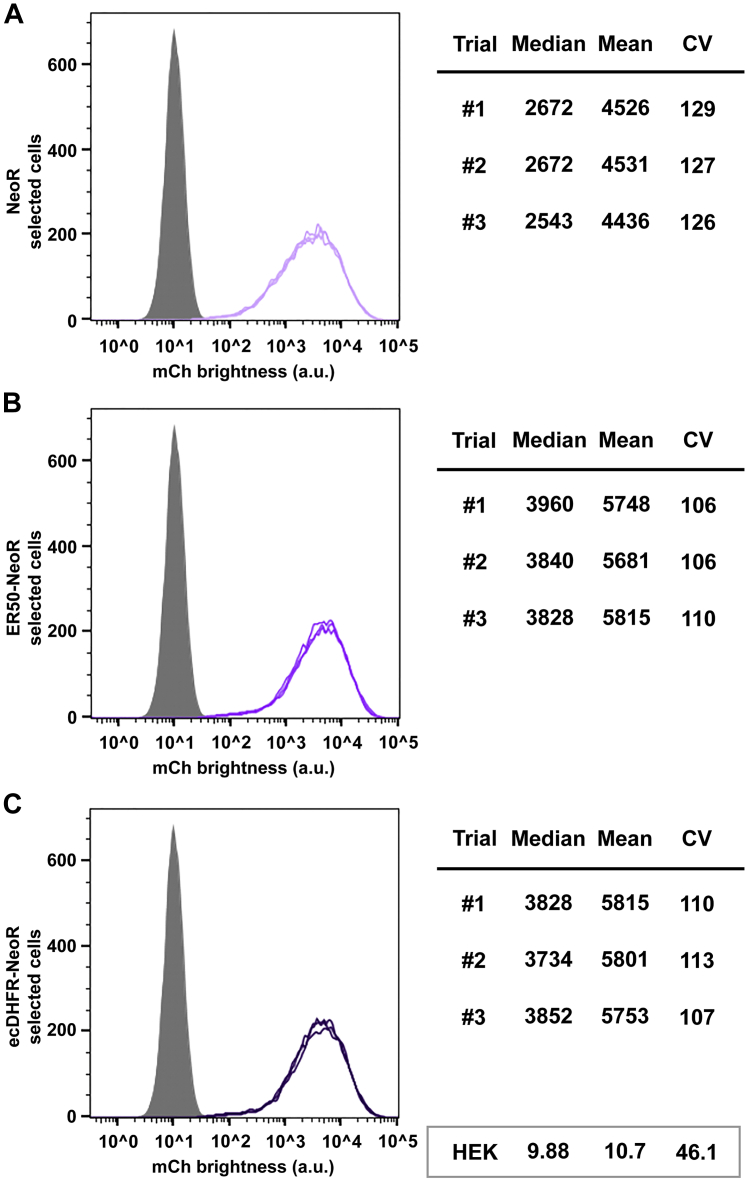
**Degron tagging has only minimal effects on NeoR-selected transgene expression.** Flow cytometry measurements of mCherry expression levels (fluorescence brightness, arbitrary units) in the polyclonal cell lines selected using transposons carrying (*A*) the untagged NeoR ORF, (*B*) the ER50NeoR ORF, and (*C*) the ecDHFRNeoR ORF. Data are shown for three technical replicates of each cell line, involving ∼20,000 independent cell fluorescence measurements for each replicate, with *gray* showing the background fluorescence of 293F cells, and median, mean, and CV from each replicate are shown to the *right*. *Light lavender* shows the average data from the NeoR-selected cell line, *medium lavender* shows the average data from the ER50NeoR-selected cell line, and *dark lavender* shows the average data from the ecDHFRNeoR-selected cell line. Data from 293F cells are shown in the *box* at the *bottom right* of the figure. ecDHFR, *Escherichia coli* dihydrofolate reductase; ER50, estrogen receptor 50.

A similar question arose from our analysis of the geneticin-resistant cell lines (Fig. 6, *A*–*C*). Consistent with our previous report (19), we found that the NeoR-selected cell line displayed mCherry levels that were lower and more homogeneous that those selected by the BleoR, PuroR, and HygR markers, and higher only than the BsdR-selected cells (Table 1). However, degron tagging had only a minimal effect on mean mCherry expression, increasing it by only 30%, and effecting only a slight drop in CV values, from 126 to ∼110.

### ER50BleoR-based selection has a preferential effect on exosome engineering

**Figure 7:**
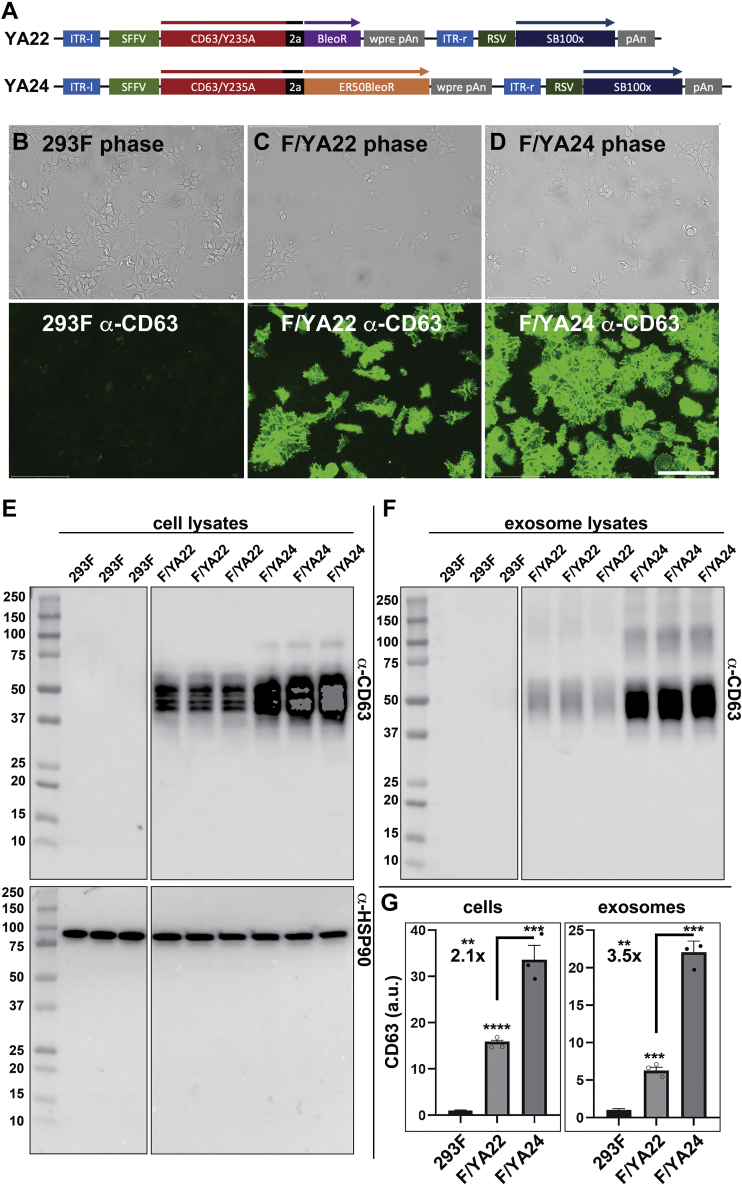
**ER50BleoR selects for higher expression and improved exosome engineering.***A*, line diagrams of Sleeping Beauty transposon vectors YA22 and YA24, which drive the expression of CD63/Y235A linked to the BleoR and ER50BleoR antibiotic resistance proteins, respectively. It should be noted that these bicistronic ORFs were expressed from the spleen focus-forming virus (SFFV) long terminal repeat (LTR), which appears to drive slightly higher transgene expression from integrated transgenes than the CMV enhancer/promoter elements (19). *B*–*D*, immunofluorescence micrographs showing anti-CD63 fluorescent antibody staining of (*B*) 293F cells, (*C*) the zeocin-resistant 293F-derived cell line YA22, and (*D*) the zeocin-resistant 293F-derived cell line YA24. *Top panels* are brightfield images, and *bottom panels* are anti-CD63 immunofluorescent images collected at the same exposure time for all three samples. The bar represents 150 μm. *E*, immunoblots of cell lysates interrogated using (*upper panel*) a monoclonal antibody specific for CD63 and (*lower panel*) an antibody-specific for HSP90. In an effort to accurately convey the difference in CD63 expression levels between 293F, F/YA22, and F/YA24 cells, we present an overexposed image of the immunoblot in this figure, though we used a nonsaturated exposure for subsequent quantification. *F*, immunoblot of equal proportions of exosomes collected from the same triplicate cultures as in (*E*), demonstrating that high-level expression of CD63/Y235A results in elevated levels of exosome-associated CD63 proteins. *G*, *bar graph* showing the amount of CD63 in cell and exosome lysates, with *bar height* denoting the average, error bars representing the standard error of the mean, *asterisks* denoting *p* value significance (∗∗<0.005, ∗∗∗<0.0005, and ∗∗∗∗<0.00005), and individual data points shown as points. Differences between the F/YA22 and F/YA24 samples were 2.1× for cell-associated CD63 and 3.5-fold for exosome-associated CD63. Numerical values were obtained by quantification of nonsaturated exposures of each immunoblot. CMV, cytomegalovirus; ER50, estrogen receptor 50; HSP90, heat shock protein 90.

The primary question arising from these studies is whether use of the ER50BleoR marker dives improved cell engineering, and more specifically, an improvement in exosome engineering (34, 35, 36, 37, 38). Toward this end, we assembled a pair of Sleeping Beauty transposon vectors designed to drive the chromosomal integration of a single transgene that expresses a bicistronic ORF encoding (i) CD63/Y235A (a mutant form of CD63 that display sixfold higher budding than WT CD63 (21)); (ii) a viral 2a peptide (28); and (iii) either the BleoR or ER50BleoR proteins (Fig. 7*A*). These vectors were transfected into 293F cells, which were then incubated in nonselective media for 2 days, followed by addition of zeocin to select for transgenic cell lines. Two weeks later, all zeocin-resistant clones from each transfection were pooled, creating the cell lines F/YA22 (BleoR based) and F/YA24 (ER50BleoR based). These cell lines were expanded under selection for an additional 2 weeks and then examined for the expression and vesicular secretion of CD63.

Immunofluorescence microscopy confirmed that the F/YA22 and F/YA24 cell lines did indeed express far more CD63 than the parental 293F cell line (Fig. 7, *B*–*D*). As for the extent of this increase, immunoblot analysis of cell and exosome fractions collected from all three cell lines (Fig. 7, *E*–*G*) revealed that F/YA22 cells contained ∼15-fold more CD63 (n = 3; *p* = 0.00000013), whereas F/YA24 cells contained ∼30-fold more CD63, relative to 293F cells (n = 3; *p* = 0.00044) (Fig. 7, *E* and *G*). The 2.1-fold higher level of CD63 expression seen for the ER50BleoR-selected cell line (n = 3, *p* = 0.0045) is consonant with the 2.3-fold increase observed for mCherry expression, indicating that the ER50BleoR drives higher recombinant protein expression, even for proteins as dissimilar as the soluble cytoplasmic mCherry and the polytopic membrane protein CD63/Y235A.

In addition to its utility for cell engineering, our data indicate that this marker may be of even greater utility for exosome engineering. Specifically, we observed that F/YA22-derived exosomes contained sixfold more CD63 than 293F-derived exosomes (n = 3; *p* = 0.00038) but that F/YA24-derived exosomes contained 22-fold more CD63 than 293F cells (n = 3; *p* = 0.00051). This 3.5-fold increase in the loading of an exosomal cargo protein (n = 3; *p* = 0.0005) raises the intriguing possibility that exosome cargo protein loading operates preferentially at the very highest levels of exosome cargo protein expression.

### Cy5-labeled CP05 peptide binds 293F cells in a CD63-independent manner

It has been reported that a short peptide, CP05 (NH2-Cys-Arg-His-Ser-Gln-Met-Thr-Val-Thr-Ser-Arg-Leu-COOH), binds specifically to the extracellular domain of CD63, and moreover, that this peptide can be used to dock a diverse array of other peptides and oligonucleotides on the surface of exosomes, imbuing them with specific biological activities and tissue tropisms (18, 39, 40, 41). These reports raise the interesting possibility that the exosomes released by F/YA24 cells, which contain 20-fold more CD63, might be the ideal type of exosomes for CP05-based exosome engineering.

**Figure 8:**
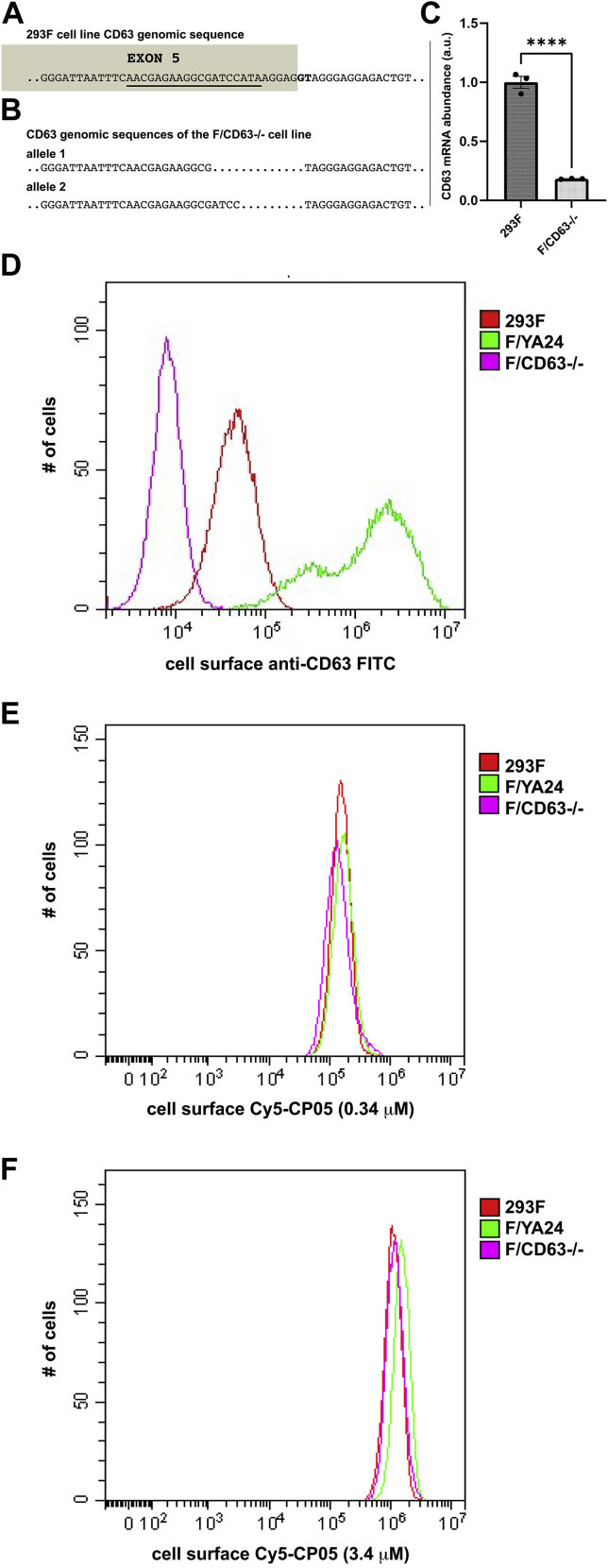
**CP05-Cy5 displays similar binding to 293F, F/CD63**^**−/−**^**, and F/YA24 cells.***A*, CD63 genomic DNA sequence in the vicinity of the Cas9/gRNA target site. *Shaded sequence* corresponds to the 3′ end of exon 5, whereas the *unshaded sequence* corresponds to the 5′ end of intron 5. *Underlined sequence* denotes the gRNA target site. *B*, DNA sequence of alleles 1 and 2 in the F/CD63^−/−^ cell line, resulting from Cas9/gRNA-mediated gene editing. *C*, CD63 mRNA abundance in 293F and F/CD63^−/−^ cells, as determined by qRT–PCR. *D*, flow cytometry histograms of (*purple*) F/CD63^−/−^ cells, (*red*) 293F cells, and (*green*) F/YA24 cells, each stained with the same FITC-labeled anti-CD63 monoclonal antibody. *E* and *F*, flow cytometry measurements of (*purple*) F/CD63^−/−^ cells, (*red*) 293F cells, and (*green*) F/YA24 cells stained with the CP05-Cy5 peptide (*D*) at a concentration of 0.34 μM of CP05-Cy5 peptide and (*E*) at a concentration of 3.4 μM of CP05-Cy5 peptide. gRNA, guide RNA; qRT–PCR, quantitative PCR.

In anticipation of using CP05 peptides to engineer exosomes, we sought to create a CD63^−/−^ cell line that could be used as a negative control for CP05-based engineering studies. Toward this end, we transfected 293F cells with recombinant Cas9 protein mixed with a CD63-specific guide RNA (gRNA) that targets the 3′ end of fifth common exon of the CD63 gene (5′-AACGAGAAGGCGATCCATA**AGG**-3’; protospacer adjacent motif site is given in bold) (Fig. 8*A*). The transfected cells were grown for several days (to allow for CD63-deficient cells to allow for turnover of pre-existing CD63 mRNA and protein) and then processed for fluorescence-activated cell sorting (FACS) using a fluorescently tagged anti-CD63 antibody, with individual CD63-deficient cells sorted into distinct wells of a 96-well plate. After 2 weeks of culture, numerous SCCs were interrogated by quantitative RT–PCR to identify cell lines that had a significant reduction in CD63 mRNA abundance, as many of the most severe mutations in human genes (*e.g.*, spice site, nonsense, and frameshift mutations) result in mRNA destruction by the nonsense-mediated RNA decay machinery (42). Candidate CD63^−/−^ cell lines were then interrogated by sequence analysis of genomic DNA in the vicinity of the Cas9/gRNA cut site. The 293F/CD63^−/−^ cell line used in the remainder of this study has two mutant CD63 alleles, both of which had deletions that removed the splice donor site at the 5′ end of intron 5 (Fig. 8*B*). These mutations preclude the proper splicing of the CD63 gene and appear to have induced nonsense-mediated RNA decay turnover of their cognate mRNAs, as seen in the approximately sevenfold reduction in total CD63 RNA abundance observed for this cell line (Fig. 8*C*). Our data cannot, however, exclude the possibility that a small amount of C-terminally truncated CD63 proteins might be expressed in this cell line, though these CD63 proteins would lack its C-terminal 54 amino acids, including the last 18 amino acids of the second extracellular loop and the entirety of the fourth transmembrane domain and cytoplasmic tail.

To determine whether these mutations led to a drop in the cell surface expression of CD63, we interrogated the 293F/CD63^−/−^ cell line, the parental 293F cell line, and the F/YA24 cell line by flow cytometry, using a commercially available fluorescently tagged monoclonal anti-CD63 antibody. The average staining intensity for the 293F/CD63^−/−^ cell line (Fig. 8*C*, *purple line*) was approximately fivefold lower than we observed for the WT 293F cell line (Fig. 8*C*, *red line*) and ∼200-fold lower than we observed for the F/YA24 cell line, which expresses high levels of CD63/Y235A (Fig. 8*C*, *green line*). Thus, while we cannot exclude the possibility that CD63^−/−^ cells express some small amount of a truncated CD63 protein, it clearly displayed less cell surface CD63 than these other cell lines, even though we failed to include the preferred control of a matched isotype staining in these experiments.

With these three cell lines in hand, we next tested the foundation of our planned CP05-based exosome engineering platform and asked whether a fluorescently tagged CP05 peptide would bind the surface of these cell lines in a CD63-dependent manner. Specifically, we synthesized the peptide NH2-CRHSQMTVTSRL(K/Cy5)-amide, resuspended it in cell-labeling buffer, incubated it with all three cell lines, washed the cells, and then interrogated each by flow cytometry. Much to our surprise, we found that this peptide displayed strong binding to the surface of all three cell lines, evident here in the strong Cy5 fluorescence detected for all three cell populations (Fig. 8*D*). Furthermore, when we repeated these experiments using a 10-fold higher concentration of CP05, we observed a 10-fold increase in the cell surface labeling of all three cell lines (Fig. 8*E*). Taken together, these results are consistent with prior observations showing that the CP05 peptide binds biological membranes but provide additional clarity to those results by showing that CP05 binds to cell membranes in a CD63-independent manner.

## Discussion

**Figure 9:**
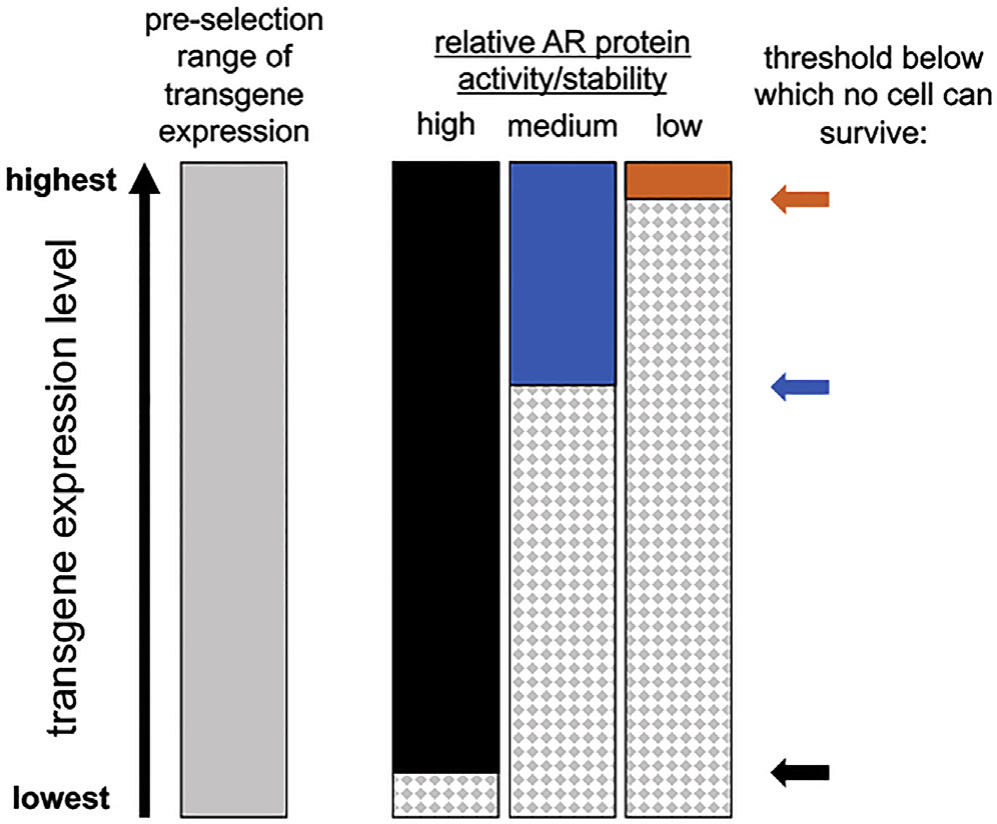
**Schematic representation of how choice of AR gene affects transgene expression.***Gray bar* represents the range of transgene expression within the entire population of transgenic cells in a transfected cell population, prior to addition of a selective antibiotic. *Black*, *blue*, and *orange bars* represent the range of transgene expression in polyclonal antibiotic-resistant cell lines selecting using AR proteins that have high, moderate, or low activity/stability, respectively. *Black*, *blue*, and *orange arrows* denote the threshold of transgene expression below which no cell can survive. *Hatched bars* represent the population of transgenic cells that will perish after the addition of selective antibiotic. AR, antibiotic resistance.

We previously established that choice of AR gene is a major factor in determining the level of transgene expression in antibiotic-resistant cell lines (19). The data presented in the present study confirm our prior observations while also providing new empirical support for our operating hypothesis. Specifically, it appears that use of the shortest-lived and least-active AR proteins yield cell lines with the highest and most homogeneous levels of transgene expression (Fig. 9), thus explaining why AR proteins fused proteasome-targeting degrons (22) selected for higher levels of linked transgene expression. Furthermore, we observed this for two of the most well-characterized degrons, the ER50 and ecDHFR destabilization domains, which have the added feature of being conditionally stabilized by small molecules (4-hydroxytamoxifen and trimethoprim, respectively) (23, 24, 25), raising the possibility that transgene expression might be further tuned by carrying out antibiotic selection in the presence of different concentrations of these drugs.

While the degron-tagging approach was generally successful, the magnitude of the degron-induced increase in transgene expression varied dramatically, from a high of the 610% increase observed for degron-tagged BsdR, to the low of the 24% increase observed for degron-tagged HygR. We do not yet know why different AR proteins display differential sensitivity to degron tagging. However, several factors indicate that reaction mechanism may be a contributing factor. For example, BleoR is the only AR protein that is not an enzyme, does not act catalytically, and instead inactivates its cognate antibiotics (*i.e.*, zeocin) by chelating them in complexes with a 1:1 stoichiometry (43). Given this nonenzymatic stoichiometric mode of action, degron tagging of the BleoR protein is predicted to have a relatively direct effect on linked transgene expression, and this is borne out by the fact that the ER50BleoR selected for approximately twofold higher levels in the linked expression of two unrelated recombinant proteins, mCherry and CD63/Y235A.

As for the four other AR proteins, they all encode an enzyme of one activity or another but differ rather dramatically in their requirement for essential cosubstrates, and in the nature of these cosubstrates. For example, BsdR encodes a blasticidin deaminase that acts by a simple hydrolysis reaction mechanism, with no known binding to any cosubstrate or cofactor present in the cell. This may render the BsdR protein particularly sensitive to degron tagging, as there is no other factor available to stabilize the protein against proteasomal degradation. On the opposite end of the scale are the HygR and NeoR proteins, both of which bind ATP because of their phosphotransferase reaction mechanisms. Without any data, it is pure speculation to suggest this, but it is at least formally possible that the presence of ATP may stabilize these proteins against degron tagging–induced turnover, much as small molecules bound by the ER50 and ecDHFR proteins stabilize them against proteasomal turnover. A similar possibility exists for the puromycin acetyltransferase encoded by PuroR, which uses a different metabolite as cosubstrate, acetyl-CoA.

Regardless of why degron tagging has more pronounced effects on some markers than on others, our data clearly show that a degron-tagged BleoR gene selected for higher levels of linked transgene expression than any of the other AR genes we tested, untagged or degron tagged. As for whether we can develop other AR genes that select for even higher levels of linked recombinant protein expression, it is an open question. After all, our operating hypothesis is that the choice of AR gene has no effect on the intrinsic levels or range of transgene expression, but rather, is made manifest by the killing of cells that do not express enough of the transgene to render cells resistant to the antibiotic (Fig. 9). This hypothesis predicts that there is a finite limit to the extent to which AR gene engineering will improve the expression of linked transgenes. In fact, our data suggest that this limit is already being approached in the case of the BleoR marker, as one degron-tagged form, ER50BleoR, selected for a twofold increase in average expression whereas tagging BleoR with the more restrictive degron, ecDHFR, failed to yield any rapidly growing colonies (23, 24, 25). If this hypothesis is correct, the ER50BleoR marker may represent a starting point for identifying and optimizing the other variables that limit the expression of recombinant proteins.

While ER50BleoR may be approaching the limit to which the BleoR protein can be improved, our data indicate that there is still significant room for improvement of the four other AR genes. Specifically, we see no reason why these selection systems cannot be improved to at least the level attained by ER50BleoR (Table 1). This suggests that there should be some way to improve puromycin selection by ∼3.4-fold higher than ecDHFRPuroR, improve hygromycin selection ~4.4-fold more than ecDHFRHyg, improve blasticidin selection by ~4.7-fold more than ecDHFRBsdR, and improve geneticin/G418 selection by ~6.4-fold higher than ecDHFRNeoR.

### ER50BleoR-mediated exosome engineering

Our research group shares the same desire for improved cell engineering as every other group that makes use of transgenic eukaryotic cells. However, our interest in exosome biology led us to test whether the improved ER50BleoR marker might have an even stronger effect on exosome engineering. Our data indicate that it does, as the approximately twofold higher level of CD63/Y235A expression led to an even larger ∼3.5-fold increase in the exosomal secretion of CD63/Y235A. We do not know the reason for this preferential impact on the exosomal loading of CD63/Y235A, as a purely stochastic effect would predict that the amount of CD63/Y235 loaded into exosomes would be proportional to the amount of CD63/Y235A expressed in the cell. The one exception to this model is if the budding of CD63/Y235A is hindered by a saturable retention that can by bypassed at the highest levels of CD63 expression.

### CD63-independent binding of cell membranes by CP05-Cy5

CD63 has long been known as an exosome marker protein (44). To identify a CD63-specific ligand, and therefore a way to selectively label exosomes, Gao *et al.* (41) carried out a phage display screen using the purified second extracellular loop of CD63 as bait. This screen yielded a short peptide (NH2-CRHSQMTVTSRL-COOH) as a candidate CD63-binding ligand. Subsequently, this peptide has been used for a technique known as “exosome painting” in which CP05 peptides are used to noncovalently attach a variety of functional and tropism-altering macromolecules to the outer surface of exosomes (19, 40, 45, 46, 47). In the context of these results, our development of cells and exosomes that display significantly higher levels of CD63 seemed a logical partner to the CP05 technology. However, in our reading of the articles that have been published on the CP05 peptide, we were unable to identify any data on the affinity of the CP05 peptide for recombinant CD63 protein and in fact could find no direct evidence of CP05–CD63 interaction of any kind. In addition, we were unable to find any evidence that the CP05 peptide requires the presence of CD63 for its membrane-binding activity.

In light of these considerations, our observation that the CP05 peptide bound equally well to F/CD63^−/−^ cells, to WT 293F cells, and to 293F cells that express 40-fold higher levels of surface CD63 is not in conflict with any prior published data of which we are aware. They are, however, in stark conflict with the hypothesis that CP05 binds to exosome membranes in a CD63-dependent manner. While it is formally possible that some unknown variable causes nonspecific binding of the CP05 peptide used in our study, the simplest explanation for our results is that CP05 peptides bind biological membranes in a nonspecific fashion. Furthermore, nonspecific binding of the CP05 peptide to the surface of biological membranes is entirely consistent with its chemical structure (CRHSQMTVTSRL), which in just 12 amino acids displays three positively charged side chains (R2, H3, and R11), four hydroxylated side chains (S4, T7, T9, and S10), and two polar side chains (C1 and Q5). In short, the CP05 peptide is a highly polar polycationic peptide nearly ideally suited for nonspecific binding to biological membranes, which are known to have a nearly unsaturable capacity for binding polar polycationic polymers. In conclusion, the CP05 peptide may be a great way to “paint” the exosome surface with CP05-coupled molecules, but we are skeptical about whether this ability is due to specific CD63–CP05 binding.

## Experimental procedures

### Plasmids

Plasmids used in this study were based on the Sleeping Beauty transposon–carrying vector pITRSB (12, 19). Each of these vectors carries a single transposon–mobilized gene that is designed to express a polycistronic ORF in which (i) the coding sequence of the protein of interest is followed by (ii) codons for the porcine teschovirus 2a peptide and then (iii) the coding sequence of the antibiotic-resistance protein, either untagged or carrying an N-terminal destabilization domain. Plasmid maps were assembled using SnapGene software (Insightful Science), all coding sequences were confirmed by DNA sequence analysis, and plasmids were prepared using commercial alkaline lysis and purification kits (Promega). Plasmids were maintained and amplified in DH10B *E. coli* cells grown in ampicillin-containing Luria broth media.

### Cell culture

293F cells (catalog no.: A14528; Thermo Fisher Scientific) were grown in complete media (chemically defined media [CM], Dulbecco's modified Eagle's medium supplemented with 10% fetal bovine serum and 1% penicillin/streptomycin) at 37 °C and 5% CO_2_. For stable clone selection, 293F cells were transfected with plasmid DNAs using Lipofectamine 3000 (Thermo Fisher Scientific), incubated for 48 h in CM, and then split into selective media (CM containing the appropriate amount of cognate antibiotics [400 μg/ml G418, 20 μg/ml blasticidin, 400 μg/ml hygromycin B, 3 μg/ml puromycin, or 200 μg/ml zeocin]). Antibiotic-resistant clones were fed every 3 to 4 days in selective media until distinct drug-resistant clones were large enough to be seen by eye, typically 2 weeks. The thousands of antibiotic-resistant SCCs that arose from each transfection were then pooled to create a single polyclonal cell line from each transfection, which were then expanded for an additional 2 weeks under antibiotic selection. Cells were then processed for flow cytometry, fluorescence microscopy, exosome collection, and immunoblot.

### Flow cytometry

For measurement of mCherry fluorescence, cells were washed in Hank’s buffered saline solution (HBSS; Thermo Fisher Scientific), released from tissue culture plates using trypsin/EDTA solution, and resuspended at a concentration of ∼1 × 10^7^ cells per ml in HBSS containing 0.1% fetal bovine serum at 4 ^°^C. Cell suspensions were maintained on ice, diluted to a concentration of 1 × 10^6^ cells per ml, and examined for mCherry fluorescence by flow cytometry on a Guava easyCyte flow cytometer (Luminex) set to the appropriate excitation and detection wavelengths. The relative brightness was determined for thousands of individual cells in each cell line using InCyte software (Luminex) and replotted with FlowJo (Beckton Dickinson), version 10, as scatter plots, average relative brightness, and CV.

For measurement of surface CD63 abundance and Cy5-CP05 peptide binding, cells were washed in HBSS, released from tissue culture plates using trypsin/EDTA solution, and resuspended at a concentration of ∼1 × 10^7^ cells per ml in HBSS. Cell suspensions were maintained on ice, diluted to a concentration of 1 × 10^6^ cells per ml, incubated with either (i) fluorescently labeled anti-CD63 antibody (catalog no.: 353006; BioLegend) or (ii) CY5-labeled CP05 peptide (NH2-CRHSQMTVTSRL(K/Cy5)-amide; Vivitide; molecular weight = 11,910). Following the binding reaction, cells were washed five times in PBS and then examined by flow cytometry on a Beckman CytoFlex flow cytometer set at the appropriate excitation and emission wavelengths. The relative brightness was determined for thousands of individual cells in each cell line using Beckman CytExpert software (version 2.3.1.22) to calculate average brightness and CV as well as to generate scatter plots and histograms.

### Microscopy

Cells were grown overnight on poly-l-lysine–coated cover glasses. Cells were then washed in PBS and fixed in 3.7% formaldehyde in PBS for 30 min. Cover glasses were then placed cells side down on 0.050 ml of FITC-labeled anti-CD63 antibody, diluted in PBS, for 30 min (catalog no.: 353006; BioLegend), washed five times in PBS, and then fixed to glass slides using 0.007 ml of Fluoromount-G (Electron Microscopy Sciences).

### Exosome purification

293F cells and derivative clones were seeded into FreeStyle 293 Expression Medium (catalog no.: 12338-018; Gibco) at a density of 1.5 × 10^6^ cells/ml in shaker flasks in a volume of approximately one-fourth the flask volume and grown for 3 days at a shaking speed of 110 rpm. Cells and large cell debris were removed by centrifugation at 5000*g* for 15 min. The supernatant was passed through an ∼200 nm pore size diameter sterile vacuum filtration unit (SteriFlip; Millipore/Sigma) to yield a clarified tissue culture supernatant. The clarified tissue culture supernatant was then subjected to two 30 min-long spins at 10,000*g* to remove any contaminating microvesicles, followed by collection of exosomes by spinning the samples at 100,000*g* for 2 h, discarding the supernatant, and resuspending the exosomes in PBS.

### Immunoblot of cell and exosome fractions

Cells and exosomes were lysed in SDS-PAGE sample buffer, separated by SDS-PAGE, transferred to polyvinylidene difluoride membranes, and blocked for 1 h in blocking solution (5% nonfat milk in TBST (50 mM Tris–HCl, pH 8.0, 138 mM NaCl, 2.7 mM KCl, 0.05% Tween-20). The blocked membranes were then incubated overnight with primary antibody diluted into blocking solution at 4 °C, with gentle rocking. Following extensive washes in TBST, the polyvinylidene difluoride membranes were incubated with horseradish peroxidase (HRP)–conjugated secondary antibodies, again in blocking solution. The membranes were again washed extensively in TBST, followed by chemiluminescent detection of HRP using ECL Plus detection reagents (GE Healthcare). Antibodies were obtained from commercial sources (mouse monoclonal anti-CD63 antibody [catalog no.: NBP2-32830] was from NOVUS, anti–heat shock protein 90 antibody [catalog no.: sc-13119] was from Santa Cruz Biotechnology, and secondary HRP-conjugated antibodies were from Jackson ImmunoResearch).

### Creation and validation of CD63-deficient 293F cells

293F cells were transfected with purified Cas9 protein (catalog no.: A36498; Invitrogen) and a gRNA (Invitrogen) targeting the sequence 5′-AACGAGAAGGCGATCCATA[AGG]-3′ in exon 5 of the CD63 gene. Following 3 days of incubation, CD63-deficient cells were isolated by FACS using an FITC-conjugated anti-CD63 antibody (catalog no.: NBP2-32830; NOVUS) on a Sony SH800S FACS machine. Single cells were seeded into a 96-well plate and expanded until the media began to turn acidic. To identify the CD63 mutations in different SCCs, genomic DNA was extracted using DNeasy Blood & Tissue Kit (Qiagen), amplified by PCR using locus-specific primers, subcloned into a plasmid vector, expanded as single clones in bacteria, and then six individual clones were sequenced from plasmids derived from each genomic DNA PCR. To measure CD63 mRNA abundance, RNA was extracted by Quick-RNA Microprep Kit (Zymo Research), followed by reverse transcription with High-Capacity RNA-to-cDNA Kit (Applied Biosystems) and quantitative real-time PCR using SYBR Green qPCR Master Mix (Bio-Rad), gene-specific primers for CD63 mRNA (5′-CAGTGGTCATCATCGCAGTG-3′ and 5′-ATCGAAGCAGTGTGGTTGTTT-3′) and 18S rRNA (5′-CGGCGACGACCCATTCGAAC-3′ and 5′-GAATCGAACCCTGATTCCCCGTC-3′), and the CFX96 Real-Time PCR detection system (Bio-Rad). The expression of CD63 mRNA relative to 18S ribosomal RNA was calculated using the ΔΔCT method.

### Statistical analysis

Statistical analysis was performed using GraphPad Prism 8 software for Windows/Mac (GraphPad Software, Inc) or Excel (Microsoft). Flow data are reported as mean, median, and CV, whereas immunoblot data are reported as mean ± standard error, with significance determined by one-way analysis of variance.

## Data availability

All data are contained within this article.

## Conflict of interest

S. J. G. is a paid consultant for Capricor, holds equity in Capricor, and is coinventor of intellectual property licensed by Capricor. S. J. T. and C. G. are coinventors of intellectual property licensed by Capricor. Y.I. declares that he has no conflicts of interest with the contents of this article.
